# Editorial: Burnout, wellbeing and resilience of healthcare workers in the post-COVID world

**DOI:** 10.3389/fmed.2025.1679590

**Published:** 2025-09-05

**Authors:** Mansoor Malik, Kristina Weeks, Tanvi Bafna, Mohua Choudhury, Kim Walker

**Affiliations:** ^1^The Johns Hopkins Hospital, Johns Hopkins Medicine, Baltimore, MD, United States; ^2^Johns Hopkins University, Baltimore, MD, United States; ^3^The Ohio State University, Columbus, OH, United States; ^4^Indian Institute of Science Department of Electrical Engineering, Bengaluru, India; ^5^University of Aberdeen, Aberdeen, United Kingdom

**Keywords:** wellbeing, COVID-19, burnout, healthcare workers, support, resilience

*This editorial synthesizes key themes from the Frontiers in Medicine Research Topic, Burnout, wellbeing and resilience of healthcare workers in the post-COVID world, highlighting critical challenges and evidence-based solutions. The studies included in this Research Topic explore the evolving nature of burnout, the structural factors exacerbating distress, and innovative strategies to support HCWs in rebuilding a sustainable healthcare workforce*.

The COVID-19 pandemic was an unprecedented global crisis that placed extraordinary demands on healthcare workers (HCWs). Long hours, moral distress, resource shortages, and the fear of infection contributed to widespread burnout, anxiety, and depression among clinicians, nurses, and allied health professionals. As we transition into a post-pandemic world, the toll on HCWs' mental health persists, necessitating urgent systemic interventions to restore wellbeing and foster resilience.

Burnout, a syndrome characterized by emotional exhaustion, depersonalization, and reduced personal accomplishment, was endemic in healthcare even before COVID-19 (1). However, the pandemic amplified its severity. Studies indicate that nearly 50-70% of HCWs experienced burnout during peak COVID-19 surges with many reporting persistent symptoms long after the acute crisis subsided (2).

The 18 studies in this Research Topic represent a diverse global perspective on healthcare worker burnout, with research teams from at least 8 different countries contributing data and analysis. The majority of studies originate from middle income countries including China, Jordan, United Arab Emirates and Iran. Several studies originated from high income countries such as the United States, United Kingdom, Sweden and Spain.

These studies paint a concerning yet actionable picture of healthcare worker wellbeing in the post-pandemic era. Across multiple countries and healthcare settings, researchers found persistently high rates of burnout and mental health challenges among healthcare professionals. Nurses and frontline workers were particularly vulnerable, with moral injury and lasting psychological scars. Several studies highlighted how systemic issues like chronic understaffing, excessive workloads, and inefficient systems (especially electronic health records) exacerbated these problems. Importantly, the research demonstrates that burnout is not simply an individual failing but rather the consequence of structural deficiencies in healthcare systems worldwide. Too often burnout interventions only target the individual factors and ignore the systemic issues.

This Research Topic includes a diverse set of healthcare workers, highlighting that burnout research must expand beyond physicians to include nurses, allied health professionals, and support staff. Understanding burnout across all healthcare workers is critical, as their wellbeing directly impacts patient care quality, team dynamics, and workforce retention. Tailored interventions are needed to address role-specific challenges and sustain the entire healthcare ecosystem.

Current conceptual frameworks distinguish wellbeing, resilience, and burnout as interrelated yet distinct factors in psychological adaptation and stress management. Wellbeing is defined by dimensions of purpose, autonomy, and personal growth; resilience is characterized as positive adaptation to adversity; and burnout is linked to emotional exhaustion and depersonalization arising from stressors. The National Academy of Medicine's conceptual model of clinician wellbeing and resilience identifies three core interconnected components that collectively influence health professionals' experiences. First, the external environment encompasses broader societal and systemic factors, including healthcare policies, regulatory requirements, and public expectations that shape workplace demands. Second, the work environment focuses on organizational and team-level elements such as workplace culture, administrative burdens, leadership practices, and clinical workflow inefficiencies that directly impact daily experiences. Third, individual factors account for personal characteristics like resilience, coping mechanisms, and life circumstances outside of work that mediate how clinicians respond to stressors. Critically, the model emphasizes the dynamic interactions between these components, illustrating how systemic pressures trickle down to affect individuals while individual wellbeing reciprocally influences organizational performance. By framing clinician wellbeing as a multi-level issue requiring coordinated solutions, the model moves beyond blaming individuals and instead highlights actionable leverage points across healthcare systems to foster sustainable resilience (3).

The Research Topic identifies several promising interventions to support healthcare workers' wellbeing. Mindfulness training, peer support groups, and gratitude practices all showed measurable benefits in reducing stress and burnout symptoms. Gao et al. showed that an online multimodal psychological support program effectively enhanced the psychological wellbeing and sleep quality of new ICU staff demonstrating the potential of off-line training in managing stress and improving health outcomes during crises. Similarly, Yang et al. showed that multi-media health education could reduce nurses' workload and enhance patient satisfaction but not increase complications. The findings of this study emphasize the importance of accessible, flexible psychological support, especially in high-stress environments such as ICUs during pandemics, natural disasters, and other emergencies.

Many studies emphasized individual level resilience building and coping. Positive religious coping was associated with reduced post-traumatic stress disorder symptoms and enhanced professional quality of life among nurses (Shiri et al.), however the systematic review by Bafna et al. and the rapid review by Frias et al., both highlight the need to target institutional culture and policy changes. Previous studies on workplace culture revealed that empathetic leadership and positive team environments significantly improve retention and job satisfaction. Practical solutions like optimized scheduling, telehealth integration, and AI-assisted documentation have emerged as effective ways to reduce administrative burdens. However, we caution that individual-level interventions alone are insufficient; the most effective approaches combine personal resilience-building with organizational changes especially in their culture. In an insightful study, included in this Research Topic, Zhang et al. identified power imbalance in the hospital dual management system as the main contributor of staff turnover. This finding is important as previous studies have calculated that nurse turnover costs hospitals ~$50,000 per replacement, making wellbeing initiatives not just ethical but financially prudent investments (4).

Similarly, a more nuanced and targeted approach to different components of burnout may be needed. Gil-Almagro et al. showed that emotional exhaustion is one of the dimensions affected in the short term and intervention programs aimed at reducing anxiety and depression at times of acute stress (onset of the COVID-19 pandemic), including thought management, seem fundamental. However, depersonalization and decreased self-fulfillment do not seem to respond to the same pattern. They seem to be a long-term result of poor management of emotional exhaustion and environmental challenges (Gil-Almagro et al.).

Looking forward, this Research Topic of evidence points to several critical needs for sustaining the healthcare workforce. There is a strong consensus that policy-level changes are required, such as changes on organizational culture to prioritize safe staffing ratios, invest in team-based care models to distribute workloads, foster supportive leadership that promotes psychological safety and open communication and ensures access to mental health care. Policy-level changes, such as fair reimbursement and regulations limiting excessive work hours, are equally critical along with establishing national/international wellbeing standards. The studies also highlight important disparities, with low-resource settings facing greater challenges due to limited infrastructure. As healthcare systems continue to recover from the pandemic shocks, this Research Topic makes clear that prioritizing worker wellbeing isn't optional, it's fundamental to delivering quality patient care. The findings collectively argue for a new paradigm where healthcare worker wellbeing is embedded in institutional operations rather than treated as an afterthought.

## References

[1] Hall LH Johnson J Watt I Tsipa A O'Connor DB. Healthcare staff wellbeing, burnout, and patient safety: a systematic review. PLoS ONE. (2016) 11:e0159015. 10.1371/journal.pone.0159015 27391946 PMC4938539

[2] Shanafelt TD West CP Dyrbye LN Trockel M Tutty M Wang H. Changes in burnout and satisfaction with work-life integration in physicians during the first 2 years of the COVID-19 pandemic. Mayo Clin Proc. (2022) 97:2248–58. 10.1016/j.mayocp.2022.09.002 36229269 PMC9472795

[3] Brigham T Barden C Dopp AL Hengerer A Kaplan J Malone B. A journey to construct an all-encompassing conceptual model of factors affecting clinician well-being and resilience. NAM Perspect. (2018) 8. 10.31478/201801b32461272

[4] Waldman JD Kelly F Arora S Smith HL. The shocking cost of turnover in health care. Health Care Manage Rev. (2010) 35:206–18. 10.1097/HMR.0b013e3181e3940e 20551768

